# 일인 가구의 성별에 따른 미충족 의료현황과 관련 요인

**DOI:** 10.4069/kjwhn.2020.03.23

**Published:** 2020-03-26

**Authors:** Hyun Ju Chae, Mijong Kim

**Affiliations:** 1 Department of Nursing, Joongbu University, Geumsan, Korea; 1 중부대학교 간호학과; 2 Department of Nursing, Hannam University, Daejeon, Korea; 2 한남대학교 간호학과

**Keywords:** Family characteristics, Women, Needs assessment, Health services accessibility, 일인 가구, 여성, 의료 요구 사정, 의료 서비스 이용

## Abstract

**Purpose:**

This study was conducted to identify unmet healthcare needs among man and woman one-person households and to explore related factors by gender.

**Methods:**

Data were drawn from the 2017 Korea National Health and Nutrition Examination Survey. The final sample consisted of 820 one-person households. The statistical analysis, conducted in SPSS version 20.1, included complex sampling analysis; descriptive statistics, the chi-square test, and logistic regression.

**Results:**

The demographic and socioeconomic characteristics of women in one-person households were significantly different from those of men in one-person households. Women in single-person households were mainly in their 70s or older and married, and they tended to have a low education level, low income, and no formal occupation. Unmet healthcare needs were experienced by 17.3% of women in one-person households and 13.5% of men in one-person households, which was not a statistically significant difference (χ^2^=2.17, *p*=.139). Factors related to unmet healthcare needs were subjective health status and unmet dental care needs in single-person-household men. By contrast, having experienced impairment within the past year, stress, and unmet dental care needs were factors related to unmet healthcare needs in single-person-household women.

**Conclusion:**

As one-person households become increasingly common, more attention needs to be paid to them and our understanding of them needs to be improved. Women in one-person households, in particular, are especially vulnerable, as they experience more unmet healthcare needs.

## Introduction

### 연구 필요성

1인 가구는 1인이 독립적으로 취사, 취침 등의 생계를 유지하는 가구를 말한다[1]. 전통적으로 가족, 가구의 개념은 부부와 그 자녀로 구성된 가족의 형태를 떠올리지만, 저출산과 고령화, 취업난, 이혼·비혼 증가로 1인 가구는 이제 우리 사회에서 흔히 볼 수 있는 가구의 형태가 되었다. 혼자 사는 가구 비율은 2000년 15%에 불과했지만, 2018년 29.3%를 차지하였으며, 서울 등 일부 지역에서는 32%를 넘는 것으로 나타났다[2]. ‘장래가구추계’에 따르면 2047년에는 전국 평균 1인 가구 비율을 37.3%로 전망하였다[1]. 1인 가구가 우리 사회에서 주된 가구 유형으로 급부상함에 따라 공적인 정책이나 민간 영역에서의 의사 결정에서 1인 가구원을 새로운 주체자 또는 대상자로 고려하고 있으며 이에 따라 1인 가구의 특성이나 취약점, 건강 요구에 대해서도 심도 깊은 정보가 필요하다.

1인 가구가 형성되는 과정을 살펴보면 일반적으로 사회구조의 변화와 경제적 여건으로 인한 비자발적 동기로 인해 발생되는 경우가 많은데[3], 특히 이러한 경제적 여건과 이혼 및 사별 등으로 인해 1인 가구가 된 경우는 다인 가구에 비해 직업의 안정성 및 소득 수준, 주거 사정, 건강 상태 등이 현저히 떨어진다고 알려져 있다[3,4]. 청년, 중년, 노년층으로 구분하여 1인 가구와 다인 가구 간의 특성을 분석한 연구에 따르면 1인 가구원이 전 생애주기에서 다인 가구 구성원에 비해 낮은 건강 수준을 나타냈다[4]. 전 연령층에서 혼자 사는 가구원은 신체적 건강 수준이 낮으며, 우울감이나 자살생각 등 정신건강 측면에서도 매우 열악하였고, 특히 청년층 1인 가구원은 높은 흡연율과 음주율을 나타내 건강 관리의 필요성이 부각되었다[4,5].

한편, 1인 가구는 혼자 독립적으로 취침 등의 생계를 유지하는 가구라는 공통점 이외에는 동질적으로 보기 어려운 다양한 속성을 가진 집단이다[3-5]. 이들은 성별, 세대별로 1인 가구가 된 동기도 다양하고, 이들을 둘러싼 사회·경제적 상황도 상이하며 어려움을 겪는 문제, 사회·복지정책 수요도 다양하다[6].

국민건강영양조사 자료를 이용하여 가구 형태에 따른 과일·야채 식이 패턴을 분석한 한 연구[7]에서 1인 가구 남성은 미혼의 비율이 41.5%인 반면에 1인 가구 여성은 기혼인 경우가 90%이며, 1인 가구 남성의 가구 소득이 100만 원 미만인 경우가 42.3%인 반면 여성 1인 가구는 100만 원 미만이 70%로 여성 1인 가구에서 저소득층이 더 많았다. 나이, 가구, 소득, 김치 섭취 등을 보정한 후에도 여성 1인 가구원은 남성 1인 가구원에 비해 과일과 야채 섭취율도 더 저조하였다[7]. 1인 가구를 미혼과 기혼으로 구분하여 각각의 삶의 만족감에 관련 요인을 분석한 연구에서도 결혼 여부와 관계없이 1인 가구의 삶의 만족감은 낮았으며 특히 기혼 1인 가구는 상대적으로 더 낮은 삶의 만족감을 보였다[8].

한편, 개인이 스스로 의료 서비스를 받을 필요가 있다고 인식하거나 의료 전문가가 의료 서비스의 필요성을 판단했음에도 불구하고 필요한 의료 서비스를 받지 못하거나 지연되는 것을 미충족 의료(unmet healthcare needs)라고 정의한다[9,10]. 충족되지 않은 의료 수요가 증가하였을 때 질병의 심각성이 커지고, 합병증의 가능성이 높아지며, 건강에 취약한 인구 집단에서는 사망률에도 영향을 미칠 수 있다[5,11]. 미충족 의료는 가용성(availability), 접근성(accessibility), 수용성(acceptability)의 측면에서 그 원인을 찾아볼 수 있는데[5], 가용성 측면에서는 병원의 대기 시간이 길거나 거주지역에 의료 자원이 충분하지 않아서 필요할 때 서비스를 이용할 수 없는 경우가 포함되며, 접근성은 필요한 의료 서비스를 얻는 경제적 능력이 부족하거나 의료기관까지의 교통의 편의에 관련된 측면이다. 마지막으로 수용성 측면에서 환자가 스스로 건강 문제를 무시하거나, 어디를 가야 할지 모른다거나, 의사에 대한 반감이나 두려움 등으로 인해 필요한 의료 서비스를 받지 못하는 경우를 생각할 수 있다[5,11,12]. 또한 여성의 건강 수준은 일반적으로 동일한 연령과 환경에서 남성에 비해 취약하다는 것이 많은 연구를 통해 나타나고 있으며[6,13-15], 1인 가구에 있어서도 역시 여성의 주관적 건강 인지나 정신건강 건강 행태는 남성에 비해 취약하다고 알려져 있다[16,17].

보건과 건강 분야에서 1인 가구를 대상으로 한 선행연구를 살펴보면, 주로 1인 가구의 일반적 특성을 파악하거나[3-6] 신체적, 정신적인 건강 및 식이를 포함한 건강 행태[6-8,13,18], 그리고 의료 이용[5,11,14]을 파악한 연구가 대부분이다. 1인 가구의 남성과 여성의 성별에 따라 건강 문제나 의료 이용을 분석하여 비교한 연구는 전무한 실정이며, 선행연구에서는 1인 가구의 다양한 인구사회학적 특성을 변수별로 구분하여 파악해볼 것을 지적하고 있다[3-6]. 특히 성별의 변수는 사회경제적인 수준이나 건강의 측면에서 중요한 차이를 보이는 변수로 나타나므로[6], 보다 포괄적인 이해를 위해서는 동일한 1인 가구 집단에서도 남성과 여성을 구분하여 그 특성을 탐색할 필요가 있다.

적절한 시기에 필요한 의료 서비스를 받지 못하는 미충족 의료는 건강과 생명에 직접적인 영향을 주는 중요한 변수인데, 선행연구에서 알려진 바와 같이 여성의 신체적, 정신적, 건강 행태의 취약성이 미충족 의료라는 의료 이용의 차원에서도 여전히 존재하는지 확인할 필요가 있다. 이를 위하여 1인 가구라는 인구학적 특성 하에서 남성과 여성을 구분하여 미충족 의료에 대해 비교할 필요가 있으며, 건강 상의 불평등이 존재한다면 향후 이를 개선하기 위하여 관련 요인이 무엇인지를 파악할 필요가 있다. 따라서 본 연구는 1인 가구원을 대상으로 성별에 따라 미충족 의료의 실태와 관련 요인을 파악하고자 연구되었으며, 추후 질병의 조기 발견과 질병 예방을 위한 의미 있는 기초자료를 제공할 것으로 생각된다.

### 연구 목적

본 연구에서는 1인 가구원을 대상으로 성별에 따른 미충족 의료의 특성을 파악하고 관련된 요인을 확인하고자 하며, 구체적인 목표는 다음과 같다.

1) 1인 가구원의 성별에 따라 일반적 특성과 건강 관련 특성의 차이를 비교한다.

2) 1인 가구원의 성별에 따라 미충족 의료 및 이유의 차이를 비교한다.

3) 1인 가구원의 성별에 따라 미충족 의료의 차이를 보이는 변수를 비교한다.

4) 1인 가구원의 성별에 따른 미충족 의료 관련 요인을 파악한다.

## Methods

Ethics statement: This study was secondary analysis of an existing national dataset and the data were received in anonymous format. As such, Institutional Review Board approval was not sought but the study adhered to principles of the Helsinki Declaration.

### 연구 설계

본 연구는 1인 가구를 구성하는 구성원을 대상으로 남녀 성별에 따른 미충족 의료의 특성을 파악하고 관련된 요인을 확인하고자 수행된 서술적 조사연구로 국민건강영양조사 제7기 2차년인 2017년도 자료를 이차 분석한 연구이다.

### 연구 대상

본 연구 대상은 2017년 1월에서 12월까지 질병관리본부에서 실시한 국민건강영양조사 제7기 2차년도 조사에 참여한 총 8,127명 중에 만 19세 이상의 성인인 동시에 1인 가구원인 사람들을 대상으로 하였다. 즉, 19세 이상이며 “세대 유형은 다음 중 무엇에 해당합니까?”라는 질문에 1인 가구를 선택한 응답자를 성인 1인 가구로 분류하여 최종적으로 만 19세 이상 1인 가구원 820명의 응답자료를 분석하였다.

### 연구변수

#### 일반적 특성

본 연구에서 일반적 특성은 국민건강영양조사의 건강설문조사 중 연령, 거주 지역, 결혼 상태, 교육 수준, 가구 소득, 직업에 대한 자료를 사용하였다. 연령은 만 나이를 기준으로 19–29세, 30–39세, 40–49세, 50–59세, 60–69세, 70세 이상으로 분류하였다. 거주 지역은 도시와 시골로 분류하였고, 결혼 상태는 미혼과 기혼으로 분류하였다. 교육 수준은 초등학교 졸업 이하, 중학교 졸업, 고등학교 졸업, 대학교 졸업 이상으로 분류하였고, 가구 소득은 개방형으로 작성된 가구총소득을 월평균 가구 균등화 소득에 따라 4개의 군으로 등분하여 하, 중하, 중상, 상의 네 그룹으로 분류하였으며, 직업은 유, 무로 분류하였다.

#### 건강 관련 특성

건강 관련 특성은 국민건강영양조사의 건강설문조사 중 월간 음주율, 현재 흡연율, 주관적 건강인지, 손상, 건강검진 수진, 암 검진 수진, 치과 미충족 의료, 스트레스 인지, 우울감 경험, 자살 생각 자료를 사용하였다. 음주는 월간 음주율에서 평생 비음주와 최근 1년간 월 1잔 미만의 음주는 비음주, 최근 1년간 월 1잔 이상의 음주는 음주로 분류하였다. 흡연은 현재 흡연율에서 비흡연과 과거 흡연은 비흡연, 현재 흡연은 흡연으로 분류하였다. 주관적 건강 인지는 주관적 건강 상태에서 매우 좋음과 좋음은 좋음, 보통은 보통, 나쁨과 매우 나쁨은 나쁨으로 분류하였고, 손상은 1년간 손상 발생 여부를 ‘예’와 ‘아니오’로 분류하였다. 건강검진과 암 검진은 최근 2년 동안 건강검진과 암 검진 수진 여부를 ‘예’와 ‘아니오’로 분류하였고, 치과 미충족 의료는 최근 1년 동안 치과 진료가 필요하였으나 받지 못한 경험 유무를 ‘예’와 ‘아니오’로 분류하였다. 스트레스 인지는 스트레스 인지율에서 ‘스트레스 적게 느낌’은 ‘아니오’, ‘스트레스 많이 느낌’은 ‘예’로 분류하였다. 우울감은 2주 이상 연속 우울감 여부를 ‘예’와 ‘아니오’로 분류하였고, 자살 생각은 1년간 자살 생각 여부를 ‘예’와 ‘아니오’로 분류하였다.

#### 미충족 의료

미충족 의료는 국민건강영양조사 건강설문조사의 의료 이용 자료를 사용하였다. 미충족 의료는 최근 1년 동안 병의원(치과 제외) 진료가 필요하였으나 받지 못한 경험 유무를 ‘예’와 ‘아니오’로 분류하였고, 미충족 의료 이유는 진료가 필요하였으나 받지 못한 주된 이유를 ‘시간이 없어서’, ‘증상이 가벼워서’, ‘경제적인 이유’, ‘기타’로 분류하였다.

### 자료 수집 방법

본 연구는 국민건강증진법 제16조에 근거하여 시행하는 국민의 건강 행태, 만성질환 유병 현황, 식품 및 영양섭취 실태에 관한 법정조사인 국민건강영양조사에서 수집한 제7기 2차년도 조사자료를 이용하였다. 국민건강영양조사에서는 건강설문조사와 검진조사는 이동검진센터에서 실시하였으며 영양조사는 대상 가구를 직접 방문하여 실시하였고, 건강설문조사의 교육 및 경제활동, 이환, 의료 이용 항목, 영양조사의 전체 항목은 면접 방법으로, 건강설문조사 항목 중 흡연, 음주 등 건강 행태 영역은 자기보고 방식으로 조사하였다. 지난 1년간 필요한 의료 서비스를 받지 못하거나 늦게 받은 적이 있는가를 질문하여 미충족 의료로 간주하였다. 본 연구자들은 위와 같이 수집된 국민건강영양조사 자료를 질병관리본부 홈페이지에서 제시하는 소정의 절차를 거쳐 파일로 제공받아 이용하였다.

국민건강영양조사는 국가가 직접 공공복리를 위해 수행하는 연구조사이며 동시에 개인정보 보호법 및 통계법을 준수하여 조사자료에서 개인을 추정할 수 없도록 비식별 조치된 자료만을 제공하고 있다. 질병관리본부의 엄격한 자료 관리 하에 학술연구 등의 목적에 한해서는 해당 자료의 제한적 활용이 허용된다. 본 연구자들은 본인의 개인정보를 밝히고 학술적 목적으로 제공되는 국민건강영양조사 원시자료를 받아 이용지침에 따라 분석하였다.

### 자료 분석

본 연구는 국민건강영양조사 2017년 원시자료로 제공된 자료를 사용하였으며 자료분석은 IBM SPSS Statistics ver. 20.0 (IBM Corp., Armonk, NY, USA)을 이용하였다. 복합표본설계 요소를 반영하여 분석하도록 하는 분석지침에 따라 수행하였으며 자료의 대표성을 보장하기 위하여 층, 집락 및 해당 조사영역을 고려하여 가중치를 이용하도록 함에 따라 가중치를 부여하여 다음과 같이 분석하였다.

1) 1인 가구 남성 가구원과 여성 가구원의 일반적 특성 및 건강 관련 특성의 차이는 복합표본 교차분석을 실시하였다.

2) 1인 가구 남성 가구원과 여성 가구원의 미충족 의료 및 이유의 차이는 복합표본 교차분석을 실시하였다.

3) 1인 가구 남성 가구원과 여성 가구원의 일반적 특성 및 건강 관련 특성에 따른 미충족 의료 실태는 복합표본 교차분석을 실시하였다.

4) 1인 가구 남성 가구원과 여성 가구원의 미충족 의료 관련 요인은 복합표본 다중 로지스틱 회귀분석을 실시하였다.

## Results

### 성별에 따른 1인 가구원의 일반적 특성과 건강 관련 특성의 차이

성별에 따른 1인 가구원의 일반적 특성과 건강 관련 특성의 차이는 Table 1과 같다. 남성 1인 가구원의 연령은 20대가 24.0%로 가장 높게 나타난 반면 여성 1인 가구원은 70세 이상이 41.8%로 가장 높게 나타났다(χ^2^=148.57, *p*<.001). 결혼 상태는 남성 1인 가구원은 미혼이 62.9%로 기혼에 비해 높게 나타난 반면 여성 1인 가구원은 기혼이 74.1%로 미혼에 비해 높게 나타나 차이를 보였다(χ^2^=112.63, *p*<.001). 교육 수준에서도 남성 1인 가구원은 대학 졸업 이상이 42.8%로 가장 높게 나타난 반면 여성 1인 가구원은 초등학교 졸업 이하가 45.5%로 가장 높게 나타나 차이를 보였다(χ^2^=117.36, *p*<.001). 가구 소득은 남성 1인 가구원과 여성 1인 가구원 모두에서 ‘하’인 경우가 가장 높게 나타났으나, 남성 1인 가구원은 ‘하’ 33.5%, ‘상’ 20.4%, 여성 1인 가구원은 ‘하’ 51.4%, ‘상’ 6.0%로 여성 1인 가구원이 남성 1인 가구원에 비해 저소득층 비율이 높게 나타나 차이를 보였다(χ^2^=47.34, *p*<.001). 직업을 가진 경우는 남성 1인 가구원에서는 65.6%인데 비해 여성 1인 가구원에서는 48.7%로 성별에 따라 차이가 있었다(χ^2^=22.35, *p*= .002).

건강 관련 특성의 차이를 살펴보면 남성 1인 가구원의 경우 음주, 흡연의 비율이 77.0%, 48.0%로 여성 1인 가구원의 41.1%, 8.1%보다 높게 나타나 차이를 보였다(χ^2^=108.02, *p*<.001; χ^2^=153.68, *p*<.001). 자신의 건강을 나쁘다고 인지하는 경우가 남성 1인 가구의 경우 22.6%인데 비해 여성 1인 가구원의 경우 36.5%로 더 높은 비율로 나타나 차이를 보였다(χ^2^=22.77, *p*<.001). 치과 미충족 의료는 여성 1인 가구원이 35.3%로 남성 1인 가구원의 23%보다 높게 나타나 차이를 보였다(χ^2^=14.74, *p*<.001). 그러나 여성 1인 가구원의 경우 암 검진 수진율은 52.36%로 남성 1인 가구원의 34.3%에 비해 높게 나타나 차이를 보였다(χ^2^=25.83, *p*<001). 우울감이 있다고 응답한 비율은 여성 1인 가구원은 22.0%, 남성 1인 가구원은 14.4%로 여성 1인 가구원에서 높게 나타나 차이를 보였다(χ^2^=7.82, *p*<.001).

### 성별에 따른 1인 가구원의 미충족 의료 및 이유

미충족 의료가 있다고 응답한 비율은 여성 1인 가구원의 경우 17.3%로 남성 1인 가구원의 13.5%보다 높게 나타났으나, 이는 통계적으로 유의하지는 않았다(χ^2^=2.17, *p*=.139). 반면, 미충족 의료 이유는 남성 1인 가구원은 시간 부족이 59.5%로 가장 많고, 증상이 가벼워서가 23.0%로 두 번째로 많았으나, 여성 1인 가구원은 시간 부족이 36.8%로 가장 많고, 경제적인 이유가 25.3%로 두 번째로 많은 것으로 나타나 차이를 보였다(χ^2^=10.55, *p*=.030) (Table 2).

### 성별에 따른 1인 가구원의 일반적 특성 및 건강 관련 특성에 따른 미충족 의료 차이

미충족 의료는 남성 1인 가구원은 주관적 건강 인지, 치과 미충족 의료, 자살 생각에 따라 차이가 있었으며, 여성 1인 가구원은 주관적 건강 인지, 손상, 건강검진, 치과 미충족 의료, 스트레스 인지, 우울감, 자살 생각에 따라 차이가 있는 것으로 나타났다.

남성 1인 가구원은 자신의 건강을 나쁘다고 인지한 경우에서 미충족 의료가 가장 높게 나타났고(χ^2^=17.65, *p*=.004), 치과 미충족 의료가 있는 경우(χ^2^=18.63, *p*=.003)와 자살 생각 경험이 있는 경우(χ^2^=5.08, *p*=.030)에서 미충족 의료가 높게 나타났다(Table 3).

여성 1인 가구원은 자신의 건강을 나쁘다고 인지한 경우에서 미충족 의료가 가장 높게 나타났고(χ^2^=21.00, *p*=.001), 최근 1년간 손상이 있었던 경우 미충족 의료가 더 높게 나타났다(χ^2^=5.24, *p*=.003). 건강검진을 받지 않은 경우(χ^2^=6.42, *p*=.023)와 치과 미충족 의료가 있었던 경우(χ^2^=26.38, *p*<.001) 미충족 의료가 높게 나타났으며, 스트레스(χ^2^=23.19, *p*<.001), 우울감(χ^2^=7.73, *p*=.008), 자살 생각(χ^2^=7.76, *p*=.016)이 있었던 경우 미충족 의료가 높은 것으로 나타났다(Table 3).

### 성별에 따른 1인 가구원의 미충족 의료 관련 요인

남성 1인 가구원의 미충족 의료 관련 요인은 주관적 건강 인지와 치과 미충족 의료인 것으로 나타났다. 자신의 건강을 좋음이라고 인지한 남성에 비해 나쁨으로 인지한 남성에서 미충족 의료가 4.87배(*p*=.003) 높게 나타났으며, 치과 미충족 의료를 경험하지 않은 남성에 비해 치과 미충족 의료를 경험한 남성에서 미충족 의료가 4.63배(*p*=.001) 높은 것으로 나타났다(Table 4).

여성 1인 가구원의 미충족 의료 관련 요인은 최근 1년간의 손상 경험, 치과 미충족 의료 및 스트레스 인지인 것으로 나타났다. 최근 1년간 손상 경험이 있었던 여성에 비해 손상 경험이 없었던 여성에서 미충족 의료가 6.48배(*p*=.005) 높게 나타났으며, 치과 미충족 의료가 없었던 여성에 비해 치과 미충족 의료가 있었던 여성에서 미충족 의료가 2.86배(*p*=.001) 높게 나타났고, 인지된 스트레스가 없는 여성에 비해 스트레스를 인지한 여성에서 미충족 의료가 2.47배(*p*=.023) 높은 것으로 나타났다(Table 5).

## Discussion

본 연구에서 1인 가구는 남녀별로 인구사회적 특성에 있어서 통계적으로 유의한 차이를 보였는데, 구체적으로 여성 1인 가구는 70대 이상, 기혼, 초등학교 졸업자, 저소득층, 직업이 없는 경우가 비율이 높게 나타났다. 남성 1인 가구에서 비율이 높았던 특성으로는 20대, 미혼, 대학 이상 졸업자, 저소득층, 직업이 있음이었다. 선행연구에서 전국적인 국가 패널 데이터를 이용하여 남성과 여성별로 1인 가구와 다인 가구의 건강행위를 비교한 연구[16,17]에서도 본 연구의 일반적인 특성과 분포가 유사하여, 1인 가구가 이질적인 특성을 가지고 있다고 설명한 선행연구를 뒷받침한다[3-5]. 1인 가구 중 특히 여성 구성원들이 혼자 살고 있다는 사회적인 고립 이외에도 고령, 저소득, 저학력, 경제활동 부재 등의 특성을 가지며 사회의 관심이 필요한 취약 집단임을 알 수 있다.

본 연구에서 분석한 성별에 따른 1인 가구의 건강 행태의 차이도 주목할 만하다. 비록 1인 가구 남성에 비해 1인 가구 여성의 음주와 흡연이 비율이 상대적으로 낮게 나타났으나 1인 가구 여성이 음주를 하는 비율이 41.1%로 나타난 것은 주목해야 할 점이다. 선행연구에 따르면 청년층 1인 가구에서 흡연율과 음주율이 다인 가구에 비하여 높아 건강 관리에 대한 필요성이 부각되었다[4]. 또한 1인 가구의 음주 행태에서 여성은 19세에서 39세까지의 1인 가구가 다인 가구에 비해 폭음을 할 위험이 높았고, 문제 음주를 할 위험도 높았으며, 40세에서 64세까지의 1인 가구도 문제 음주를 할 위험이 높았다[17,19]고 보고하였던 것과도 부분적으로 일치하는 결과이다. 1인 가구가 폭음을 할 위험이 높은 이유로, 한 번 음주를 시작한 이후 가족의 요청이나 설득 등 직접적 통제나 가족 구성원으로서 지니는 책임감에 의해 음주를 줄이고자 애쓰는 등의 간접적 통제가 없거나 부족하다[17]. 이들의 위험 건강행위를 만류하거나 곁에서 조언할 인적 자원이 부족하기 때문에 음주 행태가 악화될 경우 폭음이나 고위험 음주의 가능성이 더 높다.

1인 가구에서 주관적인 건강 인지에 대한 남녀별 차이를 살펴보면 여성이 나쁨으로 응답한 경우가 남성에 비해 높게 나타나 차이를 보였다(χ^2^=2.77, *p*<.001). 1인 가구 여성 가구원 중 36.5%가 스스로를 건강하지 않다고 생각하고 있으며, 이는 남성보다 14%나 더 많은 비율이라는 연구결과는 여성 건강 측면에서 시사하는 바가 크며, 건강 관리 전문가들이 주목해야 할 사실로 추후 다각도의 분석연구가 수행되어야 할 것이다.

한편 본 연구에서 고찰한 1인 가구원의 성별에 따른 미충족 의료 비율은 남녀의 차이가 통계적으로 유의하게 차이가 나타나지는 않았으며, 여성은 17.3%, 남성은 13.5%인 것으로 나타났다. 이 결과를 선행연구와 비교하면 2007–2012년의 국민건강영양조사를 분석하여 1인 가구의 미충족 의료 경험을 28.3%로 보고한 것[5]에 비해 낮은 수준이며, 국민건강영양조사 2015년 분석연구에서 미충족 의료 경험률을 우리나라 가구 평균 12.8%, 1인 가구 14.2%로 보고한 것[20]보다 더 높은 수준이다. 전국민 의료보험과 체계화된 의료 보장체계 내에서 의료비가 지불 가능한 수준이며 대중교통이 발달되어 병원에 대한 물리적인 접근성도 높다고 평가되는 우리나라 의료 시스템에서[21], 1인 가구 여성의 17.3%는 필요할 때 진료를 받지 못하거나 지연된 의료 서비스를 받고 있다. 1인 가구 여성의 미충족 의료만을 따로 조사한 연구는 거의 전무한 실정이므로 17.3%라는 비율만 놓고서 논의를 하기에는 제한이 있고, 또한 선행연구와 조사의 시기가 다르므로 일괄적인 비교를 하기는 무리가 있으나, 일반적으로 1인 가구원은 다인 가구원에 비해 아플 때 적절한 의료 이용을 하지 못하는 경우가 많고, 통계적 유의성은 없었으나 남성에 비해 여성에서 미충족 의료가 높게 나타난 결과는 시사하는 바가 크다.

본 연구결과에 의하면 미충족 의료의 이유로 1인 가구 남성은 시간 부족을 가장 먼저 꼽았다. 이에 비해 1인 가구 여성은 시간 부족과 더불어 경제적인 이유를 미충족 의료의 이유라고 응답하였다. 이는 선행연구에서 미충족 의료의 주된 원인을 경제적 이유와 시간 이용의 어려움 등 의료시설에 대한 가용성 2가지를 주로 제시했던 것과 유사하며[5,14], 경제적인 문제와 더불어 다양한 원인에 의해 의료적 필요에 대한 미충족이 나타날 수 있고 의료기관까지의 거리나 직장에서 자리를 비우기 어려운 경우, 의료정보의 부족 등 다양한 경우를 그 원인으로 보고한 것과도 부분적으로 일치한다[21,22]. 가족과 함께 사는 경우에 비해 혼자 사는 1인 가구원은 오직 본인이 스스로의 인식으로 자신의 건강 문제를 알아채고 병원 방문을 결정해야 하므로 이를 결정하는 것이 쉽지 않다. 또한 1인 가구원은 일반적으로 경제적으로 취약하거나 고용 형태가 안정적이지 못한 경우가 많으므로[3,4], 근무 시간을 조정하여 병원의 진료 시간에 맞추거나 장시간을 대기하여 진료를 받는 것이 쉽지 않다. 따라서 가용성, 접근성, 수용성의 세 가지 측면 모두에서 1인 가구는 취약하다고 볼 수 있다. 1인 가구 여성과 다인 가구 여성의 건강 행태와 의료 이용을 비교 분석한 연구에서도 여성 1인 가구원은 미충족 의료가 많았고, 암 검진을 실시하지 않은 비율이 높았으며, 주관적 건강 상태가 좋지 않았다[23]. 현재 미충족 의료를 파악할 수 있는 조사자료가 많지 않으며, 있다 하더라도 경험 유무와 간략한 이유를 묻는 정도이므로 다양한 변수별로 구체적인 상황과 원인을 파악하여 미충족 의료를 해결할 수 있도록 하는 연구가 후속적으로 이루어져야 할 것이다.

본 연구에서 1인 가구 구성원의 미충족 의료 관련 요인에서 남성 1인 가구원은 주관적 건강 인지와 치과 미충족 의료인 것으로 나타났으며, 여성 1인 가구원은 최근 1년간의 손상 경험, 치과 미충족 의료 및 스트레스 인지인 것으로 나타났다. 남성 1인 가구원의 경우 자신의 건강을 좋음이라고 인지한 남성에 비해 나쁨으로 인지한 남성에서 미충족 의료가 4.87배 높게 나타났다. 자신의 건강 상태에 대한 주관적인 인지는 미충족 의료에 영향을 미치는 가장 주요한 관련 요인으로 보고되고 있다[22]. 자신의 건강 상태에 대한 인지와 미충족 의료에 대한 선행연구에서는 자신의 건강을 나쁘게 인지할수록 미충족 의료가 높은 것으로 보고하고 있는데[5,11,24], 이는 본 연구와 일치하는 결과이다. 자신의 건강 상태에 대한 주관적인 인식은 실질적인 건강 수준과 밀접한 관계가 있기 때문에 미충족 의료를 개선하기 위한 정책 수립 시 대상자들이 자신의 건강을 스스로 어떻게 인지하고 있는가를 파악하는 것이 필요하다[5]. 선행연구 및 본 연구에서 자신의 건강 상태에 대해 부정적으로 인식하는 경우 미충족 의료가 높은 것으로 나타났으므로 미충족 의료를 개선하기 위하여 자신의 건강 상태에 대해 부정적으로 인식하는 대상자에게 우선적으로 초점을 맞출 필요가 있음을 보여주는 것이라고 할 것이다. 또한 객관적 건강 상태가 동일하고 유사한 의료 서비스를 받은 경우에도 자신의 건강 상태에 대한 인식에 따라 미충족 의료 경험에 대한 응답이 상이할 수 있다[22]. 이는 자신의 건강 상태를 부정적으로 인식하는 경우 필요한 의료 서비스에 대한 요구도가 높을 수 있고 이로 인해 동일한 의료 서비스를 받은 경우에도 자신이 충분한 의료 서비스를 받았다고 인지하는 정도는 낮아질 수 있기 때문이라고 할 수 있다. 또한, 의료 서비스가 충족되지 못한 이유뿐 아니라 충족되지 못한 의료 서비스가 무엇이었는지에 대한 파악과 같은 추가적이고 구체적인 분석을 통해 미충족 의료 개선을 위한 구체적이고 실질적인 정책을 수립하는 것이 필요하다.

치과 미충족 의료는 남성 1인 가구원뿐 아니라 여성 1인 가구원에서도 미충족 의료와 관련된 요인으로 나타났으며, 치과 미충족 의료가 없는 남성에 비해 치과 미충족 의료가 있는 남성에서 4.63배, 치과 미충족 의료가 없는 여성에 비해 치과 미충족 의료가 있는 여성에서 2.86배 미충족 의료가 높은 것으로 나타났다. 선행연구에서 병・의원 진료 관련 미충족 의료보다 치과 미충족 의료가 더 많이 발생하며[25,26], 병・의원 진료 및 치과 치료에서 미충족 의료가 발생하는 주된 원인은 경제적인 어려움인 것으로 보고하고 있다[25]. 이는 치과 미충족 의료는 미충족 의료와도 연결될 수 있음을 보여주는 것으로, 본 연구에서 치과 미충족 의료가 있는 경우 미충족 의료가 높게 나타난 것도 같은 맥락에서 이해할 수 있을 것이다. 본 연구 및 선행연구 결과를 고려하면 미충족 의료 및 치과 미충족 의료는 서로 분리되어 있는 개념이 아니라 동일 선상에 있는 개념으로, 치과 미충족 의료가 있을 경우 미충족 의료도 있을 가능성이 높으므로 미충족 의료 관련 실태 조사 및 정책 수립 시 미충족 의료와 치과 미충족 의료를 함께 고려하는 것이 필요하다.

여성 1인 가구원의 경우 치과 미충족 의료 이외에 최근 1년간 손상 경험이 있었던 경우에서 미충족 의료가 6.48배 더 높았으며, 스트레스를 인지한 여성에서 미충족 의료가 2.47배 더 높은 것으로 나타났다. 손상 경험과 미충족 의료와의 관계를 연구한 선행연구에서 손상 경험이 있는 대상자들의 미충족 의료 경험률이 남성 12.4%, 여성 13.9%로 나타나[27], 본 연구에서의 미충족 의료 경험률(남성 13.5%, 여성 17.3%)과 유사한 수준으로 낮게 나타났다. 손상이 있는 경우 적절한 시기에 의료 이용을 하지 못하면 합병증 및 질병 중등도의 발생 가능성이 높아질 수 있다[28]. 따라서 손상 경험이 있는 경우 신체적 불편감과 같은 증상에 민감하게 반응하여 이상 유무의 확인이나 치료를 위해 의료기관을 더 자주 방문했을 가능성이 높고, 이로 인해 본 연구에서 손상 경험이 있는 여성이 손상 경험이 없는 여성에 비해 미충족 의료가 더 적었다고 생각된다. 손상 발생 후 적절한 의료 서비스를 이용하는 것은 개인 차원에서 삶의 질을 향상시키고 국가 차원에서도 경제적 손실을 줄이기 위해 필수적임을 고려하면 본 연구에서 손상 경험이 있는 경우 미충족 의료 경험이 낮게 나타난 것은 긍정적인 결과라고 할 수 있다. 그러나 손상 경험과 미충족 의료의 관계에 대한 연구는 부족한 실정이므로 이에 대해서는 추후 반복적인 연구를 통한 근거자료 축적이 필요하다고 할 것이다.

스트레스 인지와 미충족 의료 이용의 관계에 대한 선행연구에서는 인지된 스트레스가 없음을 기준으로 했을 때 인지된 스트레스가 있는 경우 미충족 의료 경험이 더 높은 것으로 보고하고 있는데[24,27], 이는 본 연구와 일치하는 결과이다. 스트레스가 많은 경우 무력감이나 좌절감을 유발하여 의료 서비스를 이용하고자 하는 의지를 저하하거나 상실하여 미충족 의료가 발생할 수 있다[27]. 따라서 스트레스 정도 및 스트레스 원인을 파악하고 스트레스의 감소 또는 해소를 위한 상담 프로그램을 제공하여 미충족 의료를 예방하는 것이 필요하다고 할 것이다. 또한 일반인의 미충족 의료보다 정신건강과 같은 특성 대상의 미충족 의료가 높게 나타나기 때문에 스트레스, 우울, 자살 생각과 같은 정신건강 유형에 따른 미충족 의료 해결을 위한 정책 수립이 요구되고 있으므로[11], 스트레스, 우울 및 자살 생각과 미충족 의료에 대해서는 추후 계속적인 연구를 통한 실질적인 정책 수립이 필요하다고 할 것이다.

또한 본 연구에서 미충족 의료 관련 요인은 치과 미충족 의료에서 남녀 모두 유의한 관계가 있었으나 이를 제외하면 남성은 주관적 건강 인지, 여성은 손상 및 스트레스 인지가 유의한 관계가 있는 것으로 나타났다. 이는 1인 가구 구성원의 미충족 의료 관련 요인에 대한 분석 시 남성과 여성을 구분할 필요가 있음을 보여주는 것이라고 할 것이다. 따라서 추후 1인 가구원의 미충족 의료 관련 연구에서는 남성 1인 가구원과 여성 1인 가구원 각각의 미충족 의료 실태 및 관련 요인을 파악하고 이를 바탕으로 남성 및 여성 1인 가구의 특성에 맞는 차별화된 정책을 수립하는 것이 필요하다.

본 연구는 다음과 같은 연구의 제한점을 갖고 있다. 가중치를 이용하여 전국을 대표하도록 자료를 처리하였음에도 불구하고, 본 연구에서 분석한 1인 가구의 표본수가 820가구로 충분하다고 볼 수 없다. 연구결과를 해석하고 일반화하는 데 이를 고려해야 할 것이다. 또한 본 연구는 미충족 의료에 대해 1개의 문항으로 질문하여 그 응답에 따라 미충족 의료 여부를 분류하여 처리하였는데, 이는 ‘의료를 받았는데 충분하게 치료받지 못함’이나, ‘질병의 초기에 신속하게 병원을 찾지 못하고 지연된 진료를 받음’ 등 대상자에게 의료 서비스가 충족되지 않은 현황을 충분하게 반영하지 못했을 가능성이 있다. 따라서 향후 보다 충분한 수의 1인 가구를 대상으로 다양한 차원의 미충족 의료를 파악하는 연구가 수행될 것을 기대한다.

## Conclusion

본 연구는 1인 가구원의 특성을 파악하고 그들의 의료 서비스 이용에 대한 실태 중 미충족 의료를 중심으로 성별에 따라 구분하여 분석하였다. 1인 가구는 남녀 각각 인구사회학적 특성에서 여성 1인 가구가 더 높은 연령, 낮은 학력, 낮은 소득 수준, 낮은 취업 수준에 더 많이 분포하였다. 여성 1인 가구원 중 17.3%는 지난 1년간 미충족 의료를 경험했다고 응답하였고, 이는 통계적으로는 유의하지 않았으나 남성 13.5%보다는 높은 수준이었다. 미충족 의료 관련 요인은 남녀 모두에서 치과 미충족 의료가 관계가 있었으나, 치과 미충족 의료를 제외하면 남성은 주관적 건강 인지, 여성은 손상 및 스트레스 인지가 유의한 관계가 있는 것으로 나타나, 1인 가구 구성원의 미충족 의료 관련 요인에 대한 분석 시 남성과 여성을 구분할 필요가 있음을 확인하였다.

본 연구의 결과를 바탕으로 간호 실무에서 증가하는 1인 가구에 대한 이해를 높일 수 있을 것이며, 남성 및 여성 1인 가구의 특성에 맞는 차별화된 건강 증진 정책을 효과적으로 수립할 수 있을 것이다. 1인 가구 남성과 여성의 미충족 의료의 원인이 각각 차이가 있었으므로 임상이나 지역사회에서 1인 가구원의 미충족 의료 감소와 건강 증진을 위한 보건교육 시 성별에 따라 교육 전략을 차별화하여 수립할 수 있을 것이다.

## Figures and Tables

**Table 1.** General and health-related characteristics of subjects (N=820)

Variable	Categories	Men (n^†^ = 333)		Women (n^†^ = 487)		χ^2^ (*p*)
		n^†^	% (SE)	n^†^	% (SE)	
Age (year)	19–29	52	24.0 (5.1)	33	14.1 (3.1)	148.57 (< .001)
	30–39	47	20.3 (3.4)	21	6.8 (1.5)	
	40–49	48	16.3 (2.2)	22	6.1 (1.3)	
	50–59	61	16.9 (2.3)	57	12.7 (1.9)	
	60–69	62	13.2 (2.4)	116	18.5 (1.8)	
	≥ 70	63	9.4 (1.3)	238	41.8 (3.1)	
Residential area	Rural	61	17.2 (5.6)	126	21.2 (3.7)	2.14 (.502)
	Urban	272	82.8 (5.6)	361	78.8 (3.7)	
Marital status	Married	169	37.1 (3.7)	412	74.1 (3.6)	112.63 (< .001)
	Unmarried	164	62.9 (3.7)	75	25.9 (3.6)	
Education	≤ Elementary school	61	11.5 (2.2)	250	45.5 (3.4)	117.36 (< .001)
	Middle school	39	10.9 (2.0)	52	11.3 (1.4)	
	High school	102	34.8 (5.2)	79	19.6 (2.2)	
	≥ University	112	42.8 (5.3)	69	23.5 (3.5)	
Household income	Low	136	33.5 (4.6)	285	51.4 (3.5)	47.34 (< .001)
	Low middle	74	22.1 (2.9)	106	21.8 (2.2)	
	Upper middle	59	23.9 (3.6)	69	20.8 (2.8)	
	High	64	20.4 (3.4)	22	6.0 (1.3)	
Occupation	Yes	177	65.6 (4.2)	186	48.7 (3.3)	22.35 (.002)
	No	137	34.4 (4.2)	264	51.3 (3.3)	
Drinking	Yes	238	77.0 (2.3)	161	41.1 (3.3)	108.02 (< .001)
	No	91	23.0 (2.3)	318	58.9 (3.3)	
Smoking	Yes	156	48.0 (3.1)	29	8.1 (1.6)	153.68 (< .001)
	No	172	52.0 (3.1)	450	91.9 (1.6)	
Perceived health status	Poor	78	22.6 (2.5)	177	36.5 (2.6)	22.77 (< .001)
	Ordinary	160	52.9 (2.8)	211	48.9 (2.8)	
	Good	78	24.5 (2.6)	63	14.6 (2.0)	
Impairment	Yes	20	5.9 (1.6)	42	8.3 (1.3)	1.57 (.296)
	No	296	94.1 (1.6)	407	91.7 (1.3)	
Health examination	Yes	194	59.8 (3.3)	283	63.8 (2.3)	1.26 (.322)
	No	122	40.2 (3.3)	167	36.2 (2.3)	
Cancer screening	Yes	132	34.3 (2.9)	250	52.6 (3.0)	25.83 (< .001)
	No	184	65.7 (2.9)	200	47.4 (3.0)	
Unmet dental needs	Yes	84	23.1 (2.4)	179	35.3 (2.6)	14.74 (.001)
	No	244	76.9 (2.4)	300	64.7 (2.6)	
Stress	Yes	101	32.0 (2.7)	127	29.4 (2.6)	0.66 (.481)
	No	227	68.0 (2.7)	351	70.6 (2.6)	
Depression	Yes	57	14.4 (2.3)	112	22.0 (2.4)	7.82 (.017)
	No	270	85.6 (2.3)	366	78.0 (2.4)	
Suicidal ideation	Yes	42	9.3 (1.7)	47	9.2 (1.6)	0.01 (.959)
	No	286	90.7 (1.7)	432	90.8 (1.6)	

† Unweighted and valid frequency.

**Table 2.** Unmet medical needs and reasons for unmet medical needs (N=820)

Variable	Categories	Men (n^†^=333)		Women (n^†^=487)		χ^2^ (*p*)
		n^†^	% (SE)	n^†^	% (SE)	
Unmet medical needs	Yes	38	13.5 (1.8)	75	17.3 (2.1)	2.17 (.139)
	No	278	86.5 (1.8)	375	82.7 (2.1)	
Reasons for unmet medical needs	Have no time	22	59.5 (8.5)	19	36.8 (7.4)	10.55 (.030)
	Symptoms are mild	7	23.0 (8.5)	18	19.0 (5.0)	
	Economic reasons	6	13.5 (5.6)	21	25.3 (5.4)	
	Other	3	4.0 (2.1)	17	18.8 (4.9)	

† Unweighted and valid frequency.

**Table 3.** Unmet medical needs according to general and health-related characteristics (N=820)

Variable	Categories	Men (n^†^ = 333), % (SE)			Women (n^†^ = 487), % (SE)		
		Yes	No	χ^2^ (*p*)	Yes	No	χ^2^ (*p*)
Age (year)	19–29	15.9 (4.1)	84.1 (4.1)	1.27 (.923)	17.4 (6.5)	82.6 (6.5)	1.82 (.950)
	30–39	13.0 (4.6)	87.0 (4.6)		13.8 (9.1)	86.2 (9.1)	
	40–49	15.9 (5.6)	84.1 (5.6)		15.3 (10.2)	84.7 (10.2)	
	50–59	11.4 (4.3)	88.6 (4.3)		23.2 (6.1)	76.8 (6.1)	
	60–69	10.2 (4.5)	89.8 (4.5)		17.1 (5.0)	82.9 (5.0)	
	≥ 70	12.4 (4.0)	87.6 (4.0)		16.4 (2.9)	83.6 (2.9)	
Residential area	Rural	10.7 (2.9)	89.3 (2.9)	0.43 (.373)	17.4 (4.9)	82.6 (4.9)	0.00 (.992)
	Urban	14.1 (2.0)	85.9 (2.0)		17.3 (2.3)	82.7 (2.3)	
Marital status	Married	12.3 (2.7)	87.7 (2.7)	0.24 (.619)	17.5 (2.4)	82.5 (2.4)	0.04 (.879)
	Unmarried	14.2 (2.4)	85.8 (2.4)		16.7 (4.5)	83.3 (4.5)	
Education	≤ Elementary school	10.5 (4.6)	89.5 (4.6)	1.18 (.731)	17.3 (2.8)	82.7 (2.8)	4.29 (.371)
	Middle school	8.4 (4.4)	91.6 (4.4)		11.0 (4.8)	89.0 (4.8)	
	High school	14.1 (3.1)	85.9 (3.1)		23.8 (5.5)	76.2 (5.5)	
	≥ University	14.4 (3.0)	85.6 (3.0)		15.2 (4.5)	84.8 (4.5)	
Household income	Low	17.0 (3.7)	83.0 (3.7)	3.70 (.357)	20.6 (2.9)	79.4 (2.9)	5.71 (.281)
	Low middle	7.0 (3.6)	93.0 (3.6)		13.3 (3.9)	86.7 (3.9)	
	Upper middle	13.0 (3.8)	87.0 (3.8)		16.7 (5.5)	83.3 (5.5)	
	High	14.8 (4.2)	85.2 (4.2)		4.7 (4.6)	95.3 (4.6)	
Occupation	Yes	13.9 (2.3)	86.1 (2.3)	0.53 (.818)	18.2 (3.1)	81.8 (3.1)	0.22 (.684)
	No	13.0 (3.1)	87.0 (3.1)		16.6 (2.8)	83.4 (2.8)	
Drinking	Yes	12.9 (2.3)	87.1 (2.3)	0.31 (.664)	17.0 (3.6)	83.0 (3.6)	0.03 (.889)
	No	15.5 (4.9)	84.5 (4.9)		17.7 (2.8)	82.3 (2.8)	
Smoking	Yes	12.8 (2.7)	87.2 (2.7)	0.14 (.727)	23.4 (9.7)	76.6 (9.7)	0.88 (.477)
	No	14.2 (2.7)	85.8 (2.7)		16.9 (2.2)	83.1 (2.2)	
Perceived health status	Poor	28.3 (6.4)	71.7 (6.4)	17.65 (.004)	28.1 (4.1)	71.9 (4.1)	21.00 (.001)
	Ordinary	9.9 (2.7)	90.1 (2.7)		11.5 (2.6)	88.5 (2.6)	
	Good	7.6 (3.2)	92.4 (3.2)		10.0 (4.8)	90.0 (4.8)	
Impairment	Yes	10.4 (8.6)	89.6 (8.6)	0.17 (.736)	3.7 (2.5)	96.3 (2.5)	5.24 (.003)
	No	13.7 (1.8)	86.3 (1.8)		18.6 (2.2)	81.4 (2.2)	
Health examination	Yes	10.6 (2.2)	89.4 (2.2)	3.40 (.107)	13.9 (2.4)	86.1 (2.4)	6.42 (.023)
	No	17.8 (3.6)	82.2 (3.6)		23.3 (3.5)	76.7 (3.5)	
Cancer screening	Yes	9.2 (2.7)	90.8 (2.7)	2.56 (.118)	15.5 (2.5)	84.5 (2.5)	1.22 (.281)
	No	15.7 (2.3)	84.3 (2.3)		19.4 (3.0)	80.6 (3.0)	
Unmet dental needs	Yes	28.9 (7.0)	71.1 (7.0)	18.63 (.003)	29.9 (4.1)	70.1 (4.1)	26.38 (< .001)
	No	9.0 (2.0)	91.0 (2.0)		10.6 (2.2)	89.4 (2.2)	
Stress	Yes	11.7 (3.5)	88.3 (3.5)	0.45 (.565)	30.9 (5.0)	69.1 (5.0)	23.19 (< .001)
	No	14.4 (2.4)	85.6 (2.4)		11.9 (2.0)	88.1 (2.0)	
Depression	Yes	21.1 (6.0)	78.9 (6.0)	2.74 (.091)	26.7 (4.7)	73.3 (4.7)	7.73 (.008)
	No	12.2 (1.8)	87.8 (1.8)		14.7 (2.2)	85.3 (2.2)	
Suicidal ideation	Yes	26.8 (8.5)	73.2 (8.5)	5.08 (.030)	32.8 (8.1)	67.2 (8.1)	7.76 (.016)
	No	12.1 (1.7)	87.9 (1.7)		15.8 (2.1)	84.2 (2.1)	

† Unweighted frequency.

**Table 4.** Factors related to unmet health needs in man one-person households (N=333)

Variable	Categories	OR (95% CI)	*p*
Perceived health status	Poor	4.87 (1.71–13.84)	.003
	Ordinary	1.30 (0.43–3.94)	.638
	Good	Reference	
Unmet dental needs	Yes	4.63 (1.81–11.79)	.001
	No	Reference	
Suicidal ideation	Yes	1.55 (0.62–3.84)	.347
	No	Reference	
	F = 7.96, *p* < .001		

CI: Confidence interval; OR: odds ratio.

**Table 5.** Factors related to unmet health needs in woman one-person households (N=487)

Variable	Categories	OR (95% Cl)	*p*
Perceived health status	Poor	2.43 (0.79–7.50)	.120
	Ordinary	1.00 (0.34–3.30)	.998
	Good	Reference	
Impairment	No	6.48 (1.74–24.11)	.005
	Yes	Reference	
Health examination	No	1.53 (0.86–2.73)	.152
	Yes	Reference	
Unmet dental needs	Yes	2.86 (1.58–5.19)	.001
	No	Reference	
Stress	Yes	2.47 (1.13–5.36)	.023
	No	Reference	
Depression	Yes	0.96 (0.45–2.27)	.924
	No	Reference	
Suicidal ideation	Yes	1.30 (0.43–3.89)	.643
	No	Reference	
	F = 8.85, *p* < .001		

CI: Confidence interval; OR: odds ratio.
